# circ_0006789 promotes cervical cancer development via the miR-615-5p/HSF1 axis

**DOI:** 10.1007/s12672-024-01012-1

**Published:** 2024-05-15

**Authors:** Wenyu Zhou, Weiwei Song, Meisong Lu

**Affiliations:** 1 Department of Gynaecology and Obstetrics, The First Affiliated Hospital of Harbin Medical University, No.199 Dazhi Street, Nangang District, Harbin, 150000 Heilongjiang China; 2 Department of Gynaecology and Obstetrics, Shenzhen Pingshan District Maternal and Child Health Care Hospital, Shenzhen, 518100 Guangdong China

**Keywords:** Cervical cancer, circ_0006789, miR-615-5p, HSF1

## Abstract

**Objective:**

Circular RNAs (circRNAs) are involved in the development of human cancers, including cervical cancer (CC). However, the role and mechanism of circ_0006789 (circSLC25A43) in CC are unclear. The purpose of this study was to investigate the functional role of circ_0006789 in CC.

**Methods:**

The expression of circ_0006789 in CC tissues and cell lines was examined by RT-qPCR. The characterization of circ_0006789 in CC cells was verified by subcellular localisation, actinomycin D assay, and RNase R assay. After circ_0006789 was knocked down in CC cell lines, the proliferation, apoptosis, migration and invasion of CC cells were assessed by CCK-8 method, flow cytometry, and Transwell assay. RIP assay, FISH assay, dual luciferase reporter gene assay and Western blot were used to investigate the regulatory mechanism between circ_0006789, miR-615-5p and heat shock factor 1 (HSF1).

**Results:**

circ_0006789 was upregulated in CC tissues and cell lines. CC cells were inhibited in their proliferation, migration, and invasion, as well as promoted to apoptosis when circ_0006789 was knocked down. It was found that circ_0006789 targeted miR-615-5p, and miR-615-5p expression was inversely correlated with circ_0006789 expression. Furthermore, HSF1 was a target gene of miR-615-5p. Furthermore, the suppressive effects on HeLa cells mediated by circ_0006789 knockdown were counter-balanced when miR-615-5p was knocked down and HSF1 was overexpressed. Mechanistically, circ_0006789 was found to promote CC development by reducing miR-615-5p and increasing HSF1 expressions.

**Conclusion:**

circ_0006789 accelerates CC development via the miR-615-5p/HSF1 axis.

## Introduction

Cervical cancer (CC) is a prevalent gynecological neoplasm with increasing incidence and mortality rates, particularly among younger individuals. It continues to be a highly perilous gynecological malignancy worldwide, particularly in developing nations, posing a significant threat to human well-being [1]. Despite substantial progress in surgical interventions, chemotherapy, and radiotherapy, the 5-year survival rate for patients remains alarmingly low [2]. The pathogenesis of CC is governed by a diverse array of molecules, and comprehending the molecular mechanisms involved is crucial.

Circular RNAs (circRNAs) possess covalent closed-loop structures [3], which do not have 5ʹ-3ʹ polarity and polyadenylate termini. Moreover, the unique circular structure of circRNAs makes them resistant to RNase and relatively stable in tissues and cells. circRNAs are aberrantly expressed in cancer cells in a tissue-specific manner, making them promising biomarkers and therapeutic targets for tumors [4–7]. Research has indicated that circRNAs are implicated in the carcinogenesis, including CC, and can function as ceRNAs by modulating the activity of miRNAs [8, 9]. Previous investigations have identified multiple dysregulated circRNAs associated with tumorigenesis and metastasis in CC, such as circ_101996 [10], circ_0018289 [11], and circ_0000228 [12]. circ_0006789 (circSLC25A43) is a circular RNA originating from the SLC25A43 gene and is known to exert a significant influence on tumor progression. Circ_0006789 was originally thought to be able to regulate the growth, migration and invasion of hepatocellular carcinoma cells by modulating the miR-1324/SOX 12 axis [13], but no studies have yet explored circ_0006789's role in CC. Various miRNAs have been implicated in CC development, either as tumor suppressors or oncogenes[14–16]. Notably, miR-615-5p has been implicated in ovarian cancer [17]. HSF1 promotes tumor progression and tumorigenesis in cancer cells by promoting their survival and escaping programmed cell death [18–20]. Also, it has been reported that HSF1 participates in the pathogenesis of CC [21].

The objective of this study was to examine circ_0006789 expression and its biological role in CC. Our findings detected a significant upregulation of circ_0006789 in both CC tissues and cell lines. Additionally, we investigated the impact of circ_0006789 on CC development and demonstrated that circ_0006789 targets and sequesters HSF1 through miR-615-5p, thereby facilitating CC progression.

## Materials and methods

### Patients and tissue samples

CC tissues (n = 150), along with adjacent normal tissues, were collected from CC patients at The First Affiliated Hospital of Harbin Medical University between January 2021 and May 2023. Histopathology biopsy confirmation was required, followed by pathology confirmation after surgery, to determine whether a patient met the inclusion criteria. None of the subjects had undergone radiation, chemotherapy, or any treatments. The specimens were stored at − 80 °C. The clinical characteristics of the patients can be found in Table 1. Furthermore, all patients provided written informed consent. This study was endorsed by the Ethics Committee of The First Affiliated Hospital of Harbin Medical University (No. 202010HS6).

**Table 1:** circ_0006789 expression and clinicopathological features

Features	circ_0006789 expression		*P*
	Low (n = 75)	High (n = 75)	
Age (years)			
≥ 45	35	45	0.1017
≤ 45	40	30	
Lymph node metastasis			
Yes	32	38	0.0623
No	43	37	
Tumor size (cm)			
< 4	44	26	0.0032*
≥ 4	31	49	
Clinical stage			
I/II	56	34	0.0002*
III/IV	19	41	

### Cell culture

Human CC cell lines (SiHa, HeLa, HCC94, and SW756) and normal cervical epithelial cells (H8) were supplied by the BeNa Culture Collection (Beijing, China). SiHa cells were cultured in DMEM (HyClone, UT, USA), while HeLa and H8 cells with RPMI 1640 medium (Thermo Fisher Scientific, MA, USA). All cells were cultured at 37 °C and 5% CO_2_ in a complete medium (Thermo Fisher Scientific) plus 10% FBS and 1% antibiotics.

### Subcellular localization

RNA was extracted from the nucleus and cytoplasm of HeLa cells utilizing the PARIS™ Kit (Thermo Fisher Scientific). The presence of circ_0006789 was identified through PCR analysis in both nucleus and cytoplasm, with U6 and GAPDH as respective nuclear and cytoplasmic controls, respectively.

### Fluorescence in situ hybridization (FISH)

FISH assay was conducted with the FISH kit (GenePharma, Shanghai, China). The Cy5- and farm-labeled probes exhibited specificity towards circ_0006789 and miR-615-5p, respectively. Nuclei were stained with 4ʹ,6-dimethyl-2-phenylindole, and images were captured with a Zeiss LSM880 NLO confocal microscope (Leica Microsystems, Germany).

### Actinomycin D experiment

By adding 2 mg/ml actinomycin D (Sigma-Aldrich), HeLa and Siha cells were incubated at 37 °C for 4, 8, 12, and 24 h. Extracted RNA was collected using HiScript II first strand cDNA synthesis kit (Vazyme, Nanjing, China) to determine the stability of circ_0006789 using PCR.

### RNAse R

The extraction of total RNA (2 μg) from HeLa and Siha cells was conducted using TRIzol reagent (Invitrogen). Subsequently, digestion was carried out at 37 °C for 10 min with 3 U/μg RNAse R (Epicentre, WI, USA). Following RNA purification with RNeasy MinElute Cleanup kit (Qiagen, Hilden, Germany), PCR was conducted to assess circ_0006789 and SLC25A43 levels.

### Cell transfection

miR-615-5p mimic/inhibitor, si-circ_0006789, and negative controls were generated by RiboBio (Guangzhou, China). pc-HSF1, an HSF1 overexpression vector, was constructed using the pcDNA3.1 vector (Thermo Fisher Scientific). pcDNA3.1 (pc-NC) served as the negative control. HeLa and SiHa cells were seeded in 6-well plates at 3 × 10^5^ cells/mL for 24 h, followed by transfection using Lipofectamine® 3000 (Invitrogen) and PCR verification.

### PCR

Total RNA was isolated from using TRIzol reagents (Thermo Fisher Scientific). circ_0006789 and HSF1 were treated with PrimeScript™ RT Master Mix (Takara, Shiga, Japan), while miR-615-5p was detected with miRNA reverse transcription kit (TaKaRa). PCR was performed using the SYBR® Premix Ex TaqTM II kit (TaKaRa) on the StepOnePlus real-time PCR system (Thermo Fisher Scientific). GAPDH and U6 were internal parameters to calculate the relative gene expression by 2^−ΔΔCt^ method. The primers are listed in Table 2.

**Table 2:** Primers

Genes	Forward primers	Reverse primers
circ_0006789	GACCCAGACCCTCTCCTTTC	CCGTTCCAGATTTTCTCCAGG
SLC25A43	CTGGAACCATCGTACAGGGG	CCCCTGGGCCTTCACTATCT
HSF1	CATGAGAATGAGGCTCTGTG	CTACGCTGAGGCACTTTTCA
miR-615-5p	ATGCAGGGTCCGAGGTATTC	GGGGGTCCCCGGTGCT
U6	CTCGCTTCGGCAGCACA	AACGCTTCACGAATTTGCGT
GAGPH	TGTGGGCATCAATGGATTT	ACACCATGTATTCCGGGTCAAT

### CCK-8 assay

HeLa cells were put into 96-well plates (2 × 10^3^ cells/wells), on which each well was supplemented with 10 μL CCK-8 solution at 0, 24, 48, and 72 h (Dojindo, Kumamoto, Japan) and assayed for 2 h at 37 °C. Absorbance at 450 nm was read on a microplate reader (Bio-Rad, CA, USA).

### Flow cytometry

Apoptosis was assessed using the annexin V-FITC apoptosis kit (Solarbio, Beijing, China). HeLa cells were seeded at 10^5^ cells/well in a 12-well plate for 48 h. Following this, the cells were trypsinized, resuspended in a binding buffer, and subsequently incubated with 10 μL annexin V-FITC and PI for a duration of 10 min. Data quantification was done with flow cytometry (Agilent, Hangzhou, China), and apoptosis rate was calculated.

### Transwell

Transwell chambers (Corning, MA, USA) were utilized for Transwell assays. For the cell migration assay, a suspension of 1 × 10^5^ HeLa and SiHa cells in serum-free medium (150 µL) was transferred to the upper compartment. Following incubation at 37 °C for 24 h, the cells on the lower compartment were fixed with 95% ethanol and stained with 0.1% crystal violet for a duration of 25 min. The cells were enumerated utilizing a microscope manufactured by Olympus, Japan. A Matrigel layer was pre-covered in the Transwell chamber to perform invasion assay.

### Dual luciferase reporter gene experiment

The CircInteractome (https://circinteractome.nia.nih.gov/) and TargetScan (https://www.targetscan.org/) biological information website predicted the potential binding sites of miR-615-5p with circ_0006789 or HSF1 3ʹUTR. circ_0006789 or HSF1 3'UTR sequences, containing either a wild-type or mutant binding sequence for miR-615-5p, were inserted into the pmirGLO vector (GenePharma). Following the Lipofectamine 3000 protocol, miR-615-5p mimic or miR-NC and the luciferase reporter were co-transfected into HeLa and SiHa cells for a duration of 48 h. Subsequently, the luciferase activity was evaluated using the luciferase reporter assay kit (Promega).

### RIP

HeLa and SiHa cells were lysed with a complete RIP lysis buffer (Millipore, MA, USA). The supernatant was co-incubated with magnetic beads bound to Ago2 (ab32381, Abcam, UK) or IgG (02–6102, Invitrogen) at 4 °C for 6 h. After eluting the immunoprecipitate of the bound bead, circ_0006789, miR-615-5p, and HSF1 were measured by PCR.

### Immunoblot

The isolation of total protein from tissues and cells was performed using RIPA lysis buffer (Beyotime, Shanghai, China), followed by quantification analysis using the BCA kit (Beyotime). Subsequently, protein samples (30 μg/lane) were separated through sodium dodecyl sulphate–polyacrylamide gel electrophoresis and subsequently transferred to a polyvinylidene fluoride membrane (Millipore). Then, the membrane was sealed with 5% skim milk for 1 h, subjected to incubation with HSF1 (4356, CST), Vimentin (ab92547, Abcam), E-cadherin (14472, CST), N-Cadherin (13116, CST), and GAPDH (5174, CST) overnight and horseradish peroxidase-labeled IgG (ab124055, Abcam) for 1 h, and developed with ECL kits (Thermo Fisher Science) in a chemiluminescent imager (Image Quant LAS4000 mini, GE Healthcare, UK). The Image J software analyzed protein bands.

### Xenotransplantation model

Five-week-old female BALB/c nude mice (Beijing Laboratory Animal Center, Beijing, China) were utilized to construct the xenotransplantation model. HeLa cells were transfected with circ_0006789 or si-NC (RiboBio, Guangzhou. China) and selected using purinomycin. Subsequently, each mouse was given a subcutaneous inoculation of stably transfected HeLa cells (5 × 10^6^ cells) into the lateral abdomen (n = 6/group). The tumor volume was assessed weekly for a duration of 4 weeks. Volume (mm^3^) = length × width2/2. Under isoflurane anesthesia, the mice were euthanized after completing the experiment. The tumor samples were weighed, and then circ_0006789, miR-615-5p and HSF1 were detected by PCR and immunoblot. Animal treatment conformed to the approval by the Animal Management and Use Committee of The First Affiliated Hospital of Harbin Medical University (No. 202012B01).

### Data analysis

All data were expressed as the mean ± standard deviation (SD) of more than 3 replicates and analyzed using GraphPad Prism 9.0 (GraphPad, CA, USA). One-way analysis of variance was employed for multiple-group differences, and Student’s t-test was used for comparing two-group differences. Chi-square test was utilized to evaluate the relationship between circ_0006789 expression and clinicopathological information. * *P* < 0.05 was considered statistically significant.

## Results

### Circ_0006789 expression and clinicopathological features

**Fig. 1:**
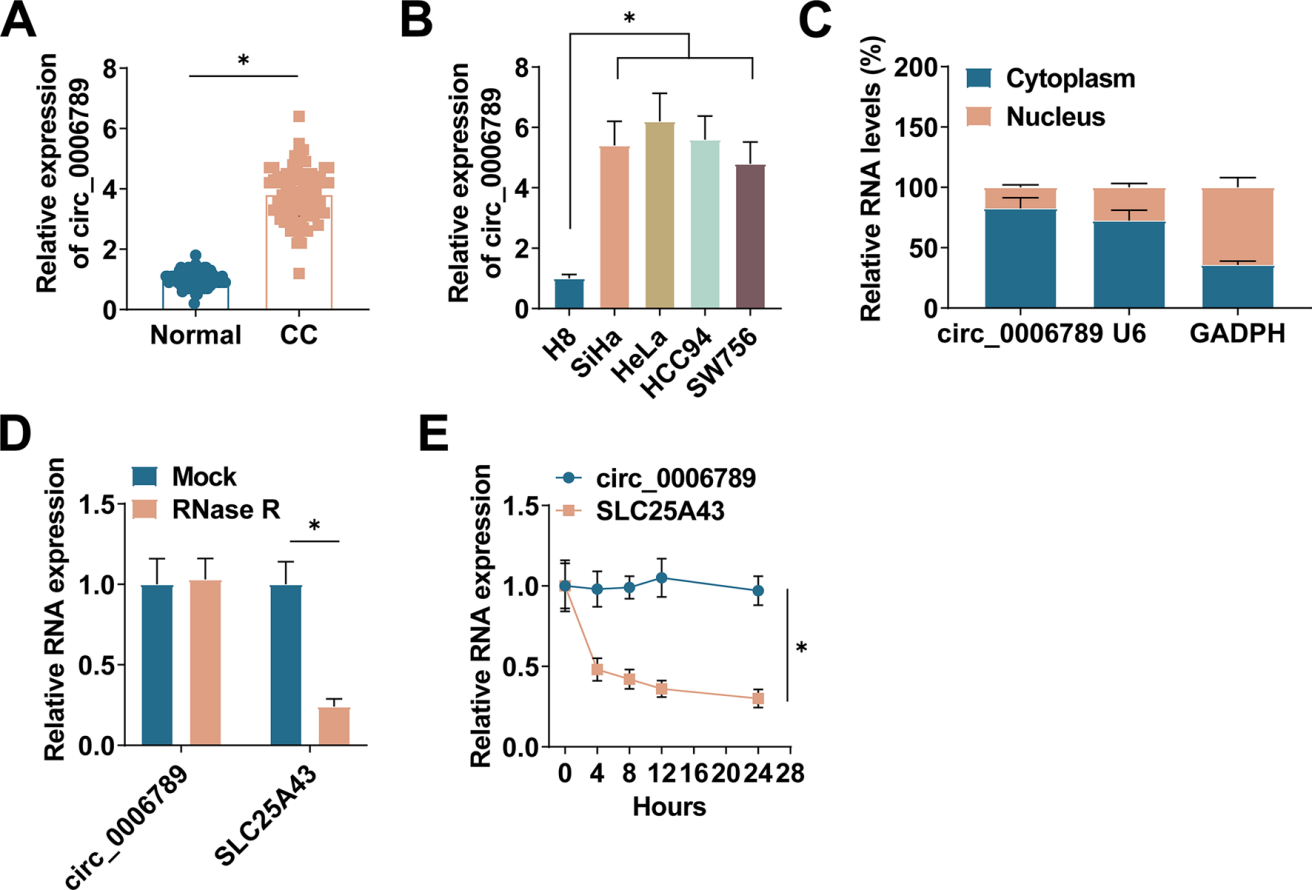
circ_0006789 expression in CC. **A** PCR measured circ_0006789 in CC and adjacent normal tissues. **B** PCR measured circ_0006789 in CC cells (SiHa, HeLa, HCC94 and SW756) and normal cervical epithelial cells (H8). **C** Nucleocytoplasmic separation experiment detected circ_0006789 in cytoplasm and nucleus. **D** RNAse R assay detected the stability of circ_0006789. **E** PCR measured circ_0006789 after actinomycin D treatment at different time points. Data are expressed as mean ± SD (N = 3), * *P* < 0.05

To detect circ_0006789 levels in CC, 150 paired patient’s tissue samples were collected. PCR data determined that circ_0006789 expression was significantly enhanced in CC tissues (Fig. 1A). circ_0006789 levels in CC cell lines (SiHa, HeLa, HCC94, and SW756) were higher than in H8 cells (Fig. 1B). The localization of circ_0006789 in cells was further examined to determine its function in CC. Cytoplasmic separation test revealed that circ_0006789 was mainly distributed in the cytoplasm of HeLa cells (Fig. 1C). RNAse R experiments demonstrated that circ_0006789 exhibited resistance to RNAse R, whereas SLC25A43 expression was significantly diminished following RNAse R treatment (Fig. 1D). This observation suggests that circ_0006789 is a circular transcript. Furthermore, when subjected to actinomycin D treatment, circ_0006789 displayed insensitivity, further supporting its circular nature (Fig. 1E). Therefore, circ_0006789 may be involved in the corresponding molecular regulation and function post-transcriptionally. Based on circ_0006789 levels, the patients were categorized into two groups: those with high circ_0006789 expression and those with low circ_0006789 expression. circ_0006789 levels were found to be correlated with tumor differentiation and TNM stage, while no significant correlation was observed with age or lymph node metastasis (Table 2).

### Knockdown of circ_0006789 significantly inhibits CC cell proliferation, migration and invasion and promotes apoptosis

**Fig. 2:**
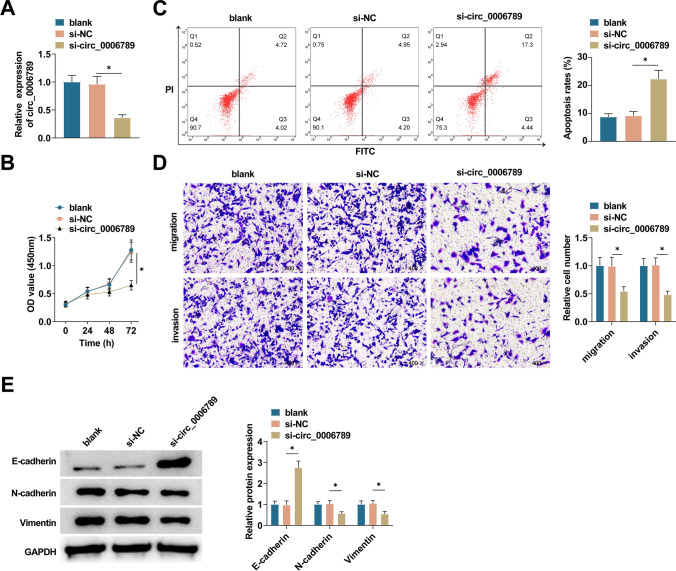
Effect of knockdown of circ_0006789 on CC cells. **A** PCR measured circ_0006789 in HeLa cells transfected with si-circ_0006789. **B** CCK-8 assay detected the proliferation of HeLa cells after transfection with si-circ_0006789. **C** Flow cytometry detected the apoptosis of HeLa cells after transfection with si-circ_0006789. **D** Transwell assay detected the migration and invasion ability of HeLa cells transfected with si-circ_0006789. **E** Immunoblot measured Vimentin, E-cadherin, and N-cadherin in HeLa cells transfected with si-circ_0006789. Data are expressed as mean ± SD (N = 3), * *P* < 0.05

HeLa cells were subjected to transfection with si-circ_0006789. Following transfection, a notable decrease in circ_0006789 expression was observed, indicating a significant efficacy of si-circ_0006789 transfection (Fig. 2A). The assessment of cell proliferation capacity was conducted using the CCK-8 method, while cell apoptosis rate was determined through flow cytometry analysis. circ_0006789 deficiency resulted in a substantial reduction in cell proliferation capacity and an enhancement in apoptosis in HeLa cells (Fig. 2B, C). Transwell assay was employed to investigate the impact of circ_0006789 on the migratory and invasive abilities of CC cells. Notably, circ_0006789 depletion resulted in suppression of migration and invasion in HeLa cells (Fig. 2D). Furthermore, immunoblot analysis was conducted to assess the influence of circ_0006789 knockdown on EMT-related proteins. It was observed that circ_0006789 knockdown elevated E-cadherin and lowered N-cadherin and Vimentin levels (Fig. 2E).

### circ_0006789 is a sponge of miR-615-5p

**Fig. 3:**
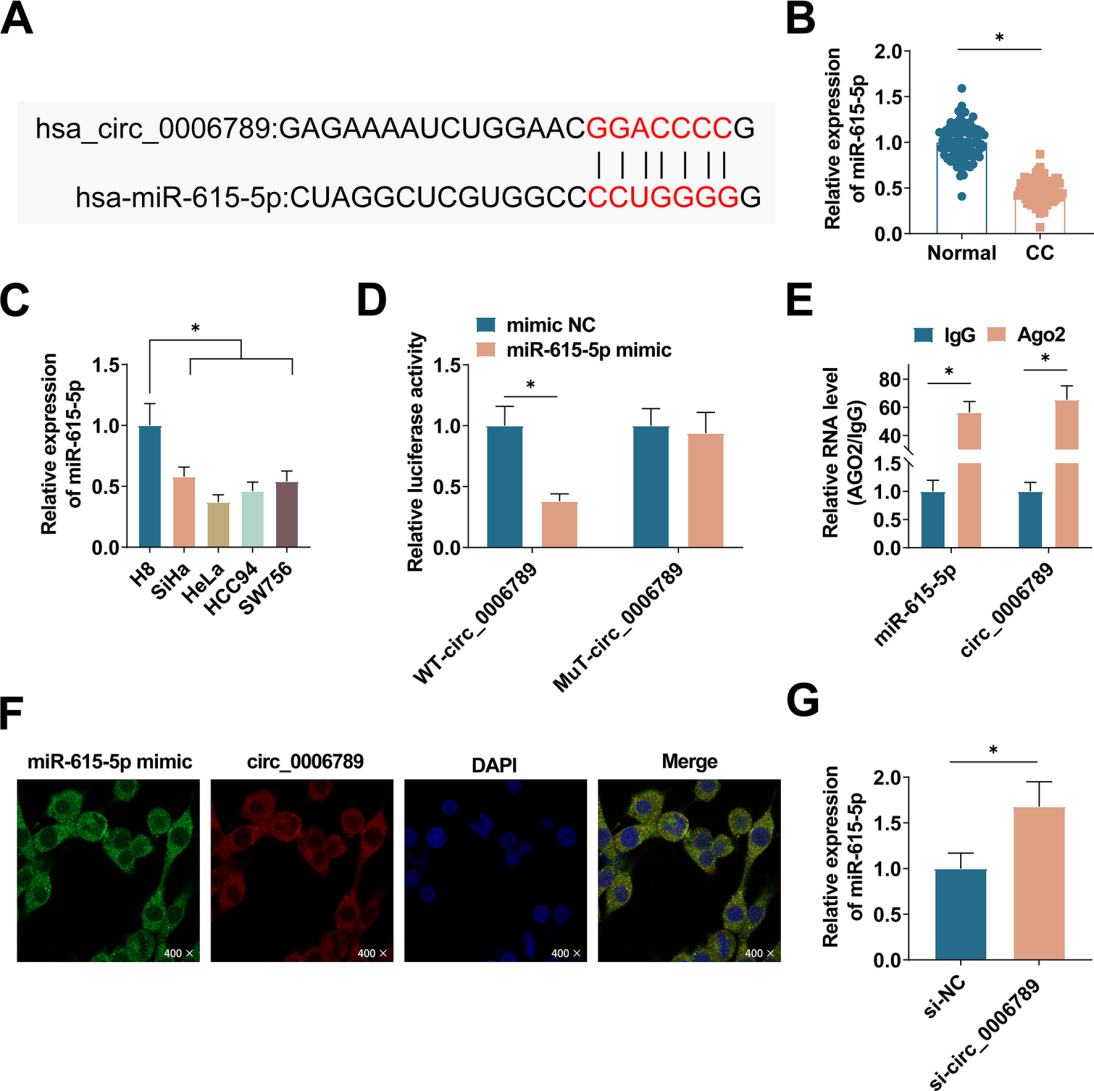
Binding relationship between circ_0006789 and miR-615-5p. **A** CircInteractome predicted the binding site of miR-615-5p to circ_0006789. **B** PCR detected miR-615-5p in CC tissues. **C** PCR detected miR-615-5p in CC cells. **D**, **E** Dual luciferase reporter gene analysis and RIP analysis verified the targeting relationship between circ_0006789 and miR-615-5p. **F** FISH detection demonstrated that circ_0006789 and miR-615-5p were co-located (400×). **G** PCR detected miR-615-5p in HeLa cells after transfection with si-circ_0006789. Data are expressed as mean ± SD (N = 3), * *P* < 0.05

miR-615-5p was selected as the predicted target miRNA, and the corresponding binding sites were predicted by CircInteractome (https://circinteractome.nia.nih.gov/) (Fig. 3A). Downregulated miR-615-5p in CC tissues and various CC cell lines was quantified using PCR (Fig. 3B, C). Furthermore, a dual luciferase reporter gene assay demonstrated that co-transfection of miR-615-5p mimic and WT-circ_0006789 lowered luciferase activity, whereas co-transfection with MUT-circ_0006789 did not alter cellular luciferase activity (Fig. 3D). Subsequently, the results of the RIP experiment revealed that circ_0006789 and miR-615-5p exhibited enrichment in the Ago2 group, thereby suggesting a direct interaction between circ_0006789 and miR-615-5p (Fig. 3E). Furthermore, FISH experiments demonstrated the co-localization of circ_0006789 and miR-615-5p in the cytoplasm (Fig. 3F). Moreover, this investigation observed an up-regulation of miR-615-5p expression through PCR analysis following the transfection of HeLa cells with si-circ_0006789 (Fig. 3G).

### Knockdown of miR-615-5p reduces the impacts of circ_0006789 deletion on CC cells

**Fig. 4:**
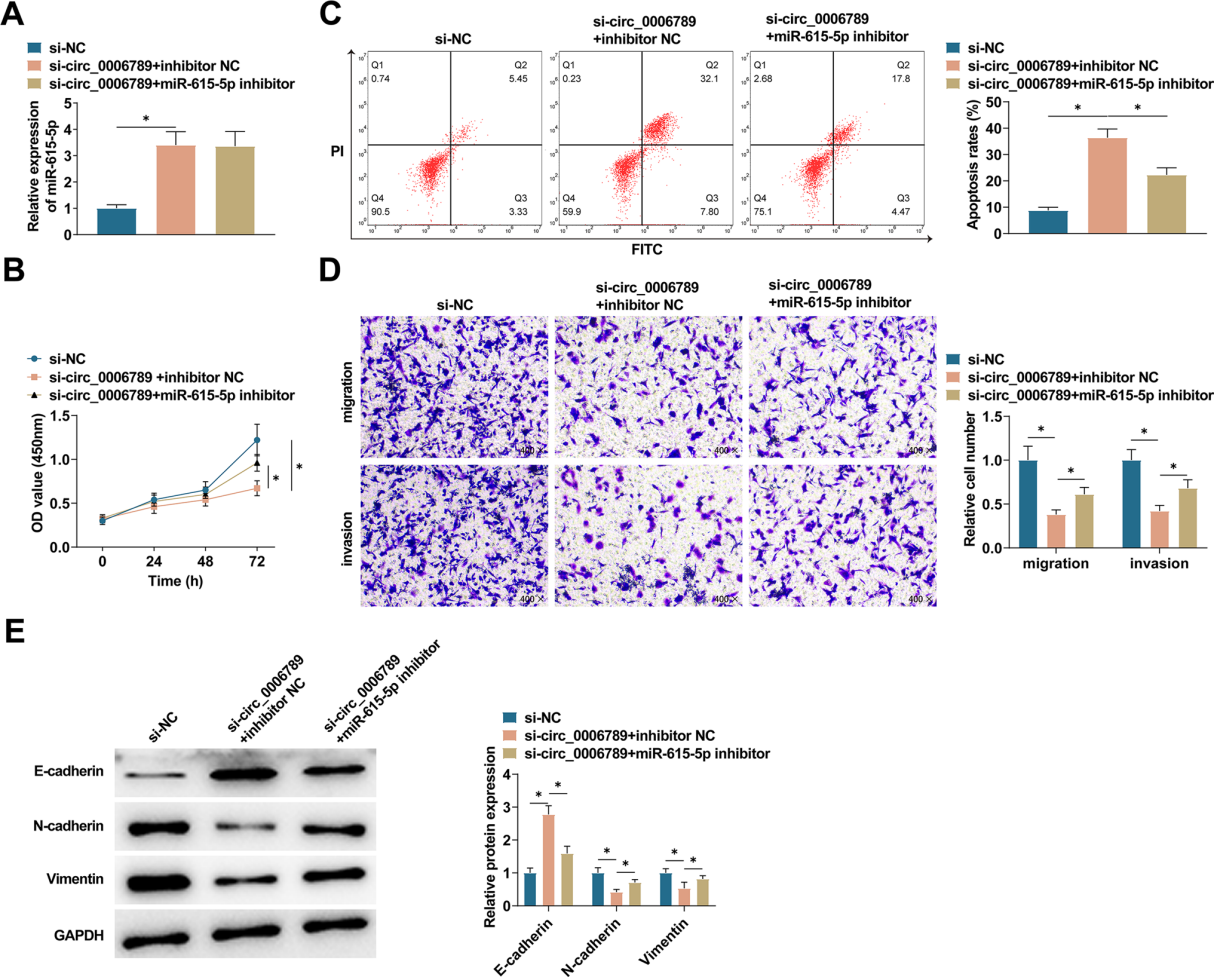
Effect of circ_0006789 regulating miR-615-5p expression in CC development. **A** PCR detected miR-615-5p after transfection in HeLa cells. **B** CCK-8 assay detected the proliferation of HeLa cells. **C** Flow cytometry detected the apoptosis of HeLa cells. **D** Transwell assay detected the migration and invasion ability of HeLa cells. **E** Immunoblot measured Vimentin, E-cadherin, and N-cadherin in HeLa cells. Data are expressed as mean ± SD (N = 3), * *P* < 0.05

HeLa cells were co-transfected with si-circ_0006789 and miR-615-5p inhibitor. PCR analysis confirmed that si-circ_0006789 forced miR-615-5p expression levels (Fig. 4A). Additionally, CCK-8 experiments demonstrated that inhibiting miR-615-5p mitigated the suppressive effect of circ_0006789 knockdown on HeLa cell proliferation (Fig. 4B). Additionally, flow cytometry indicated that inhibiting miR-615-5p counter-balanced the pro-apoptotic effect of circ_0006789 knockdown on HeLa cells (Fig. 4C). Transwell assay results indicated that inhibition of CC cell migration and invasion resulting from the knockdown of circ_0006789 was counteracted when miR-615-5p was knocked down (Fig. 4D). Immunoblot analysis revealed that knockdown of miR-615-5p lowered E-cadherin and promoted N-cadherin and Vimentin levels (Fig. 4E).

### HSF1 is the target of miR-615-5p

**Fig. 5:**
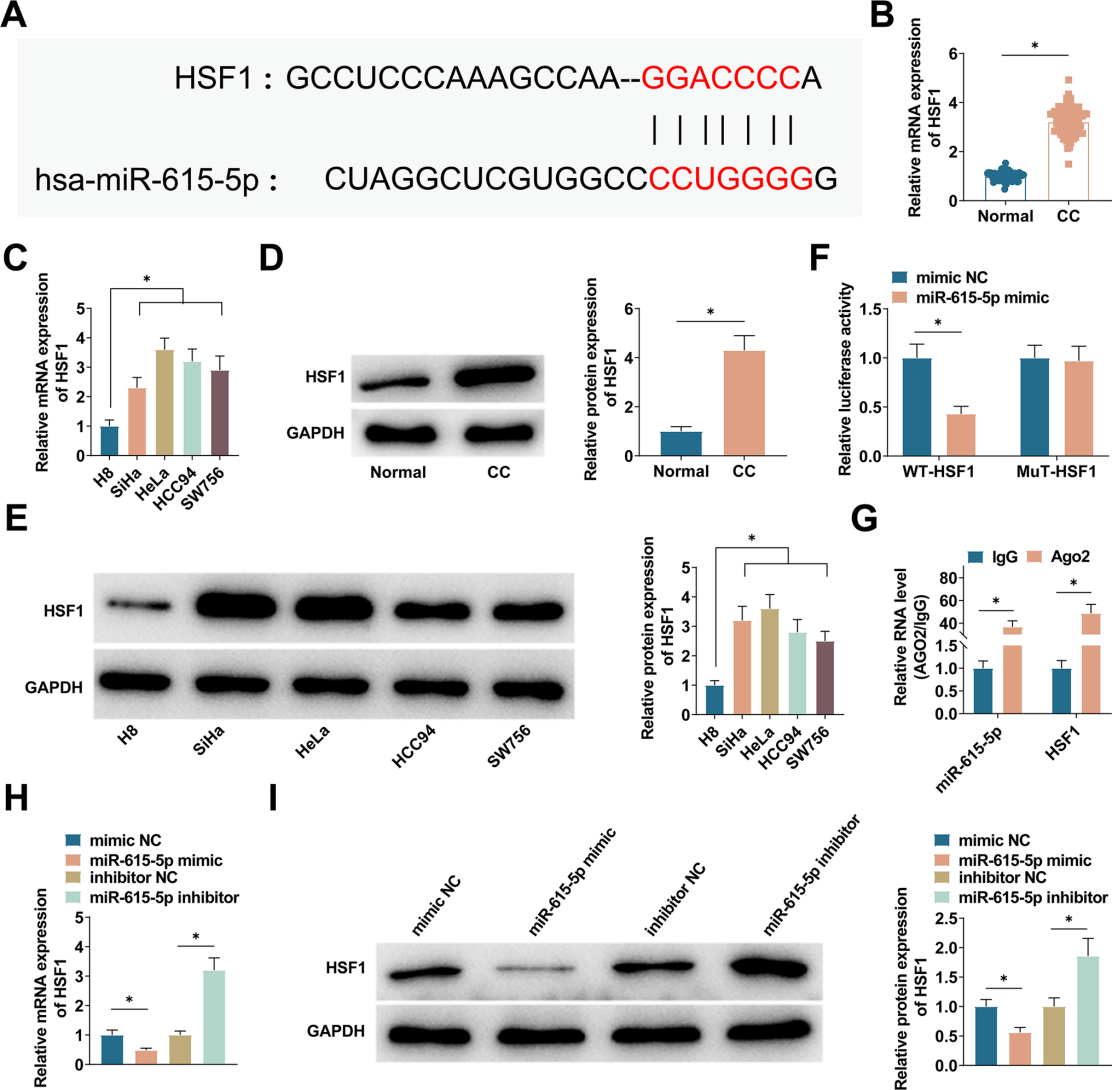
Targeting relationship between miR-615-5p and HSF1. **A** TargetScan predicted the binding sites of miR-615-5p and HSF1. **B**, **C** PCR detected HSF1 in CC tissues and cell lines. **D** Immunoblot detected HSF1 in CC tissues. **E** Immunoblot detected HSF1 in HeLa cells. **F**, **G** Dual luciferase reporter gene analysis and RIP analysis verified the targeting relationship between miR-615-5p and HSF1. **H**, **I** PCR and immunoblot detected HSF1 in HeLa cells. Data are expressed as mean ± SD (N = 3), * *P* < 0.05

TargetScan (https://www.targetscan.org/) was utilized to further study the downstream mRNA of miR-615-5p. HSF1 was selected as the predicted target mRNA, and the corresponding binding site was predicted (Fig. 5A). HSF1 expression in CC tissue and cells was detected by PCR and immunoblot. HSF1 was up-regulated in CC tissues and cells (Fig. 5B–E). Dual luciferase reporter gene experiments determined that co-transfection of miR-615-5p mimic and WT-HSF1 decreased the luciferase activity of cells, but co-transfection with MUT-HSF1 did not affect the luciferase activity of cells, indicating that miR-615-5p and HSF1 could interact in HeLa cells (Fig. 5F). Next, RIP experiment indicated that miR-615-5p and HSF1 were enriched in the Ago2 group relative to the IgG group (Fig. 5G). PCR and immunoblot results showed that miR-615-5p mimic reduced HSF1 expression levels. However, miR-615-5p inhibitor further upregulated HSF1 expression levels (Fig. 5H, I).

### Forcing HSF1 saves the suppressive impact of circ_0006789 knockdown on CC cells

**Fig. 6:**
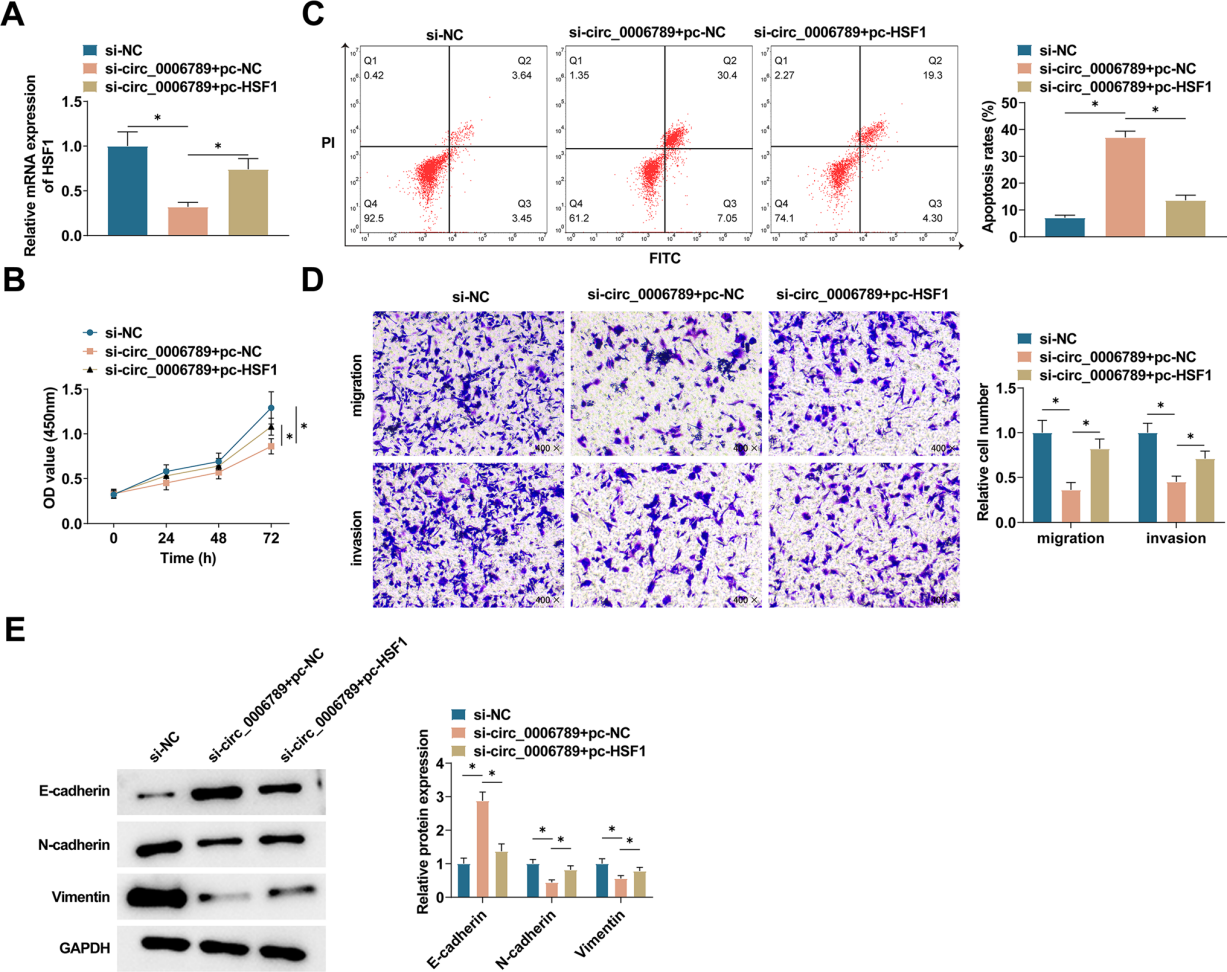
Circ_0006789 Regulation of HSF1 on proliferation, apoptotic migration and invasion of CC cells. **A** PCR detected HSF1 in HeLa cells. **B** CCK-8 assay detected the proliferation of HeLa cells. **C** Flow cytometry detected the apoptosis of HeLa cells. **D** Transwell assay detected the migration and invasion ability of HeLa cells. **E** Immunoblot measured Vimentin, E-cadherin, and N-cadherin in HeLa cells. Data are expressed as mean ± SD (N = 3), * *P* < 0.05

To further investigate the biological role of circ_0006789 in CC through its regulation of HSF1, we conducted functional rescue experiments. HeLa cells were transfected with either control siRNA, si-circ_0006789, or si-circ_0006789 along with a pc-HSF1 overexpression plasmid. PCR analysis revealed that transfection with si-circ_0006789 resulted in a decrease in HSF1 expression, whereas co-transfection with pc-HSF1 led to an increase in HSF1 expression (Fig. 6A). Additionally, CCK-8 demonstrated that elevating HSF1 mitigated circ_0006789 knockdown-mediated HeLa cell proliferation (Fig. 6B). Flow cytometry results determined that elevating HSF1 reduced the pro-apoptotic effect of circ_0006789 knockdown on HeLa cells (Fig. 6C). Transwell results indicated that the suppressive effect of circ_0006789 knockdown on CC cell migration and invasion was counter-balanced by overexpressing HSF1 (Fig. 6D). Immunoblot analysis results analyzed that forcing HSF1 up-regulated E-cadherin and decreased N-cadherin and Vimentin, reversing the effect of circ_0006789 knockdown on these proteins (Fig. 6E).

### Elimination of circ_0006789 reduces xenograft tumor growth in vivo

**Fig. 7:**
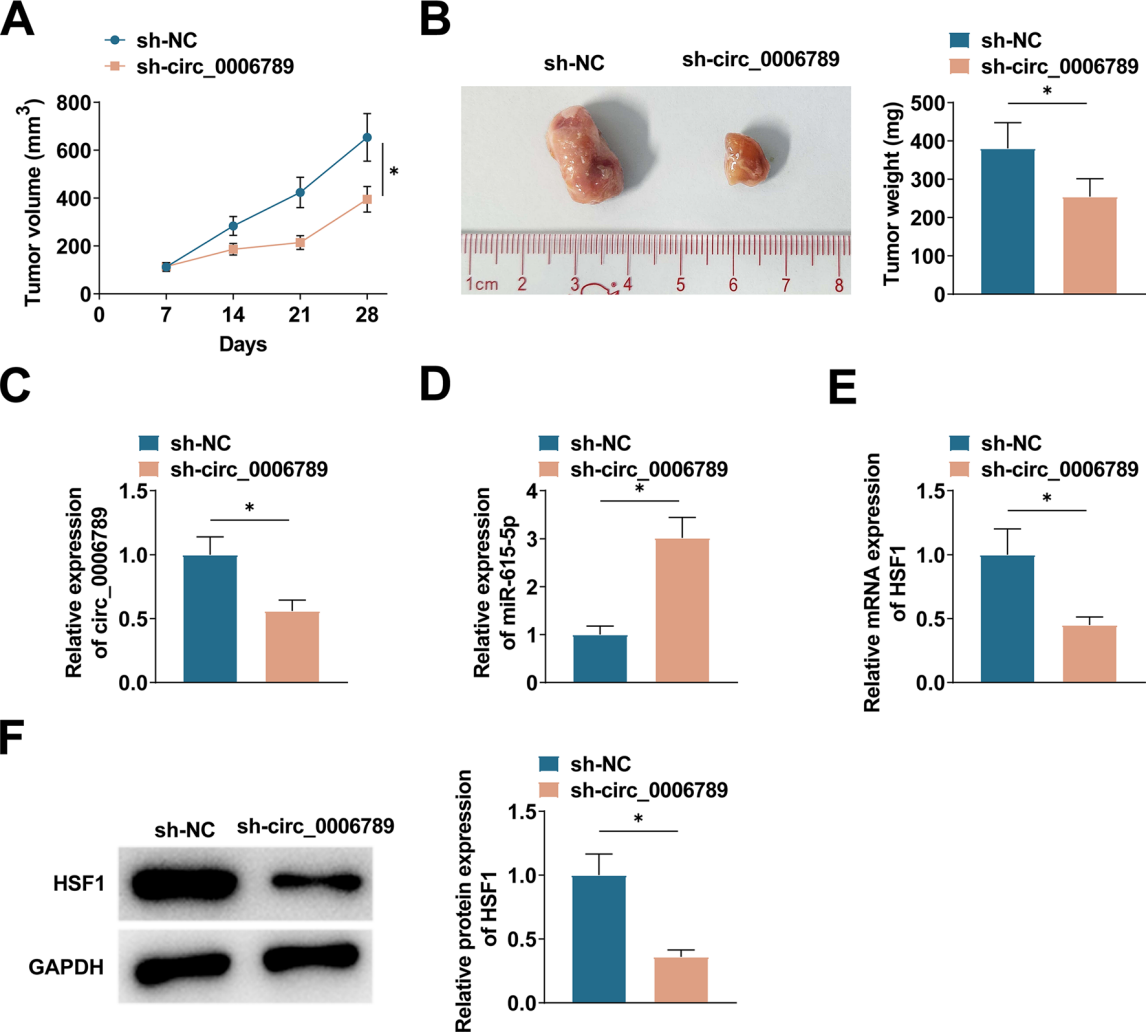
Effect of circ_0006789 on xenograft tumor growth. **A** Weekly monitoring of tumor volume; **B** Tumor weight measured at endpoint (day 28). **C**–**E** PCR detected circ_0006789, miR-615-5p, and HSFl in tumor tissues. F: Immunoblot measured HSF1 in tumor tissues. Data are expressed as mean ± SD (n = 6), * *P* < 0.05

Xenotransplantation models were established by employing stably transfected HeLa cells with si-circ_0006789 or si-NC. Weekly examination of tumor volume and measurement of tumor weight at the endpoint were conducted. The results demonstrated a reduction in tumor volume and weight in the si-circ_0006789 group (Fig. 7A, B). On day 28, the si-circ_0006789 group exhibited significant reductions in circ_0006789 and HSF1 levels, while miR-615-5p levels were enhanced (Fig. 7C–F).

## Discussion

CircRNA is considered a biomarker relevant to clinical trials and may contribute to poor prognosis in various tumors due to its stable structure.Studies have demonstrated the significant role of circRNA as a key regulator in CC [22]. For instance, circ_0000228 facilitates the malignancy of CC through the miR-195-5p/Lysyl-oxidase-like protein 2 axis [23], while circ_0000285 downregulation inhibits CC via the miR-197-3p-ELK1 axis [24]. Additionally, circ_0003221 has been found to promote CC through the miR-758-3p/CPEB axis [25]. The present study observed circ_0006789 up-regulation in both CC tissues and cell lines. Also, depleting circ_0006789 significantly hindered CC cells to proliferate, migrate, and invade, while concurrently enhancing apoptosis. These findings strongly imply that circ_0006789 possesses oncogenic properties and holds promise as a therapeutic target for CC treatment. However, the mechanism of action of CC treatment through circ_0006789 needs more exploration.

circRNA can function as a molecular sponge, exerting indirect control over gene expression through miRNA sequestration [26]. A new mechanism by which non-coding RNA regulates biological processes is suggested by the ceRNA hypothesis. The interaction between miRNA and circRNA can regulate the malignancy and aggressiveness of cancer cells [27, 28]. In this work, it is found that circ_0006789 was mainly distributed in the cytoplasm of HeLa cells, so it is speculated that circ_0006789 may have the function of ceRNA. miRNAs are an integral part of the ceRNA network and are characterized by their specific binding to the 3'UTR of target mRNAs. By blocking translation or inducing target degradation, miRNAs are involved in the regulation of gene expression at the post-transcriptional level. aberrant expression of miRNAs is also closely related to tumour development [29]. miR-615-5p is a newly identified endogenous miRNA that has been associated with the development of several cancers [30, 31]. In common with other studies, miR-615-5p was also in a dysregulated state in CC, and we found a trend of downregulation in CC tissues and cells. Meanwhile bioinformatics, dual luciferase reporter gene assay and RIP assay predicted and verified that miR-615-5p could specifically and directly bind to circ_0006789. Moreover, knocking down circ_0006789 in CC cells significantly increased miR-615-5p. Shortly, circ_0006789 participates in miR-615-5p's negative regulation in CC cells through ceRNA activity.

Multiple cellular responses related to tumorigenesis involve HSF1, including alterations in the tumor microenvironment, repairs to the genome, and other critical pathways [32]. Generally, cancer cells exhibit elevated levels of HSF1 expression [33]. Additionally, HSF1-associated non-coding RNAs have been identified in various cancer types. In the context of hepatocellular cancer cells, downregulating HSF1 reduces proliferative and anti-apoptotic abilities of tumor cells [34]. This study confirmed HSF1 overexpression in CC. HSF1 was a target gene of miR-615-5p and could be positively regulated by circ_0006789. Overexpressing HSF1 partially offset the suppressive impact of circ_0006789 knockdown on CC cells. Furthermore, a mouse xenograft model was established to elucidate the in vivo anticancer effects of circ_0006789 knockdown on CC.

The findings of this study provide insights into the molecular mechanism underlying the dysregulation of circ_0006789 in CC and demonstrate that circ_0006789 exerts a carcinogenic influence on CC through the regulation of the miR-615-5p/HSF1 axis. This study provides a new mechanism to understand the pathogenesis of CC and suggests that circ_0006789 may be a target for CC treatment. However, this study has limitations in terms of cell lines, and further studies on the roles of circ_0006789 and miR-615-5p in CC in more cell lines are needed subsequently. In addition, although the mechanism of action of circ_0006789 in CC was analyzed in this study, its potential for clinical diagnosis, prognostic assessment and as a therapeutic target needs to be validated by large-scale clinical trials.

## Data Availability

The datasets used and/or analyzed during the present study are available from the corresponding author on reasonable request.
